# Prostatic Utricle Cyst: A Clinical Dilemma

**Published:** 2013-05-10

**Authors:** Vinod Priyadarshi, Jitendra Pratap Singh, Shwetank Mishra, Mukesh Kumar Vijay, Dilip Kumar Pal, Anup Kumar Kundu

**Affiliations:** Department of Urology, Institute of Postgraduate Medical Education and Research, Kolkata. India.

**Keywords:** Prostatic utricle, Urethra, Stricture

## Abstract

Prostatic utricle cyst is a rare midline cystic lesion between the urinary bladder and the rectum, commonly associated with hypospadias. Along with its rarity, it presents a challenge in its diagnosis and proper management. We report a case of large prostatic utricle cyst that was managed conservatively.

## INTRODUCTION

Cystic enlargement of prostatic utricle, a vestigial remnant of mullerian duct, is a rare condition in males. It is present in up to 4% and 1% in newborns and adults respectively [1]. In some cases, prostatic utricle is markedly enlarged and present as a cystic lesion in perineum or in pelvic cavity posing diagnostic dilemma. We present a case of large prostatic utricle cyst associated with urethral stricture.

## CASE REPORT

A 15-year-old boy presented with dysuria for the last 3 months. He had mid penile hypospadias that was repaired in early childhood. The meatus was at ventral surface of glans and of adequate calibre. His laboratory investigations were within normal limits. Uroflowmetry confirmed poor flow while ultrasonography suggested 30 gm prostate with a midline hypoechoic cyst extending posterior to bladder into pelvic cavity. Bladder had normal wall thickness but had 40 ml as residual urine. Retrograde and voiding urethrography showed dilated penile urethra and distal bulbar urethral stricture with contrast filling in a blind ending tubular structure posterior to the bladder, arising at the level of prostatic urethra and extending beyond the level of bladder neck (fig. 1, 2). This was not palpable per abdomen. Cystourethroscopy revealed a passable stricture in distal bulbar urethra and an opening at the verumontanum ending into a blind cavity. Dorsal onlay buccal mucosa graft (BMG) urethroplasty done for the strictured segment but no treatment was offered for the prostatic utricle cyst. After urethroplasty alone, flow improved, post-void residual urine decreased and patient improved symptomatically. Patient kept on regular follow-up foe last two years and planned for surgical excision of utricle in future only if it becomes symptomatic. 

**Figure:**
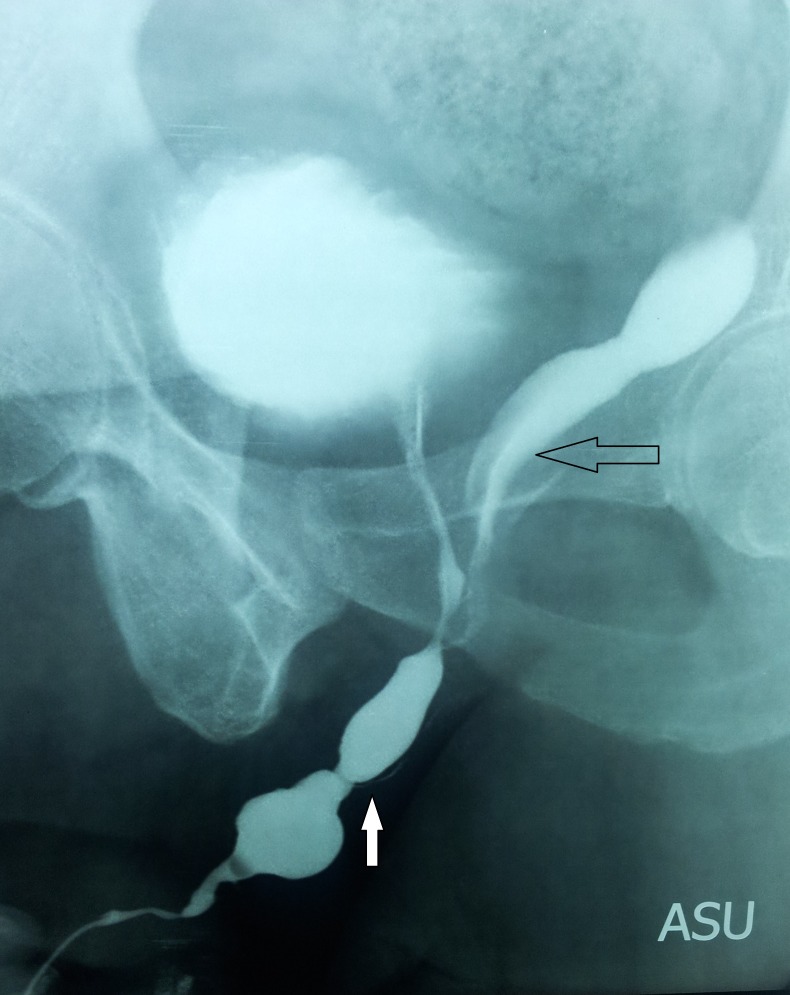
Figure 1: Ascending urethrogram: Horizontal arrow showing utricle cyst, solid arrow showing distal bulbar stricture

**Figure:**
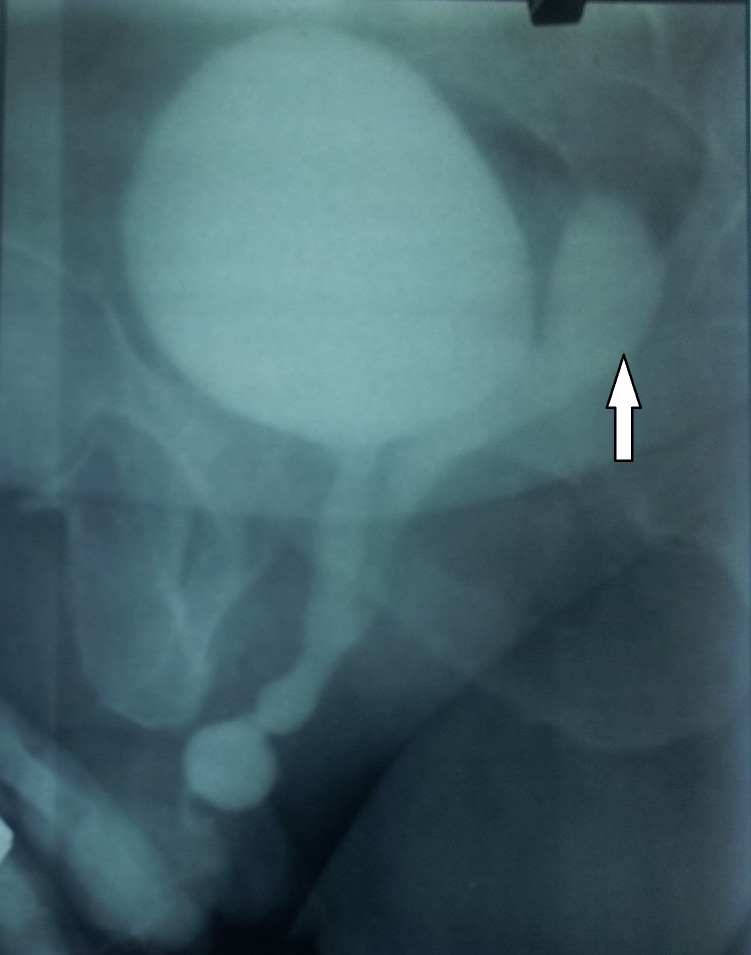
Figure 2: Voiding Cysturethrogram showing utricle cyst posterior to bladder and communicating with prostatic urethra

## DISCUSSION

Prostatic utricle is a remnant of mullerian duct in males which often present as a sac with a slit like orifice at the apex of verumontanum that projects upward and backward into the substance of the prostate [2]. The incidence of prostatic utricle cyst is 11% to 14% in association with hypospadias or intersex anomalies and up to 50% in the presence of perineal hypospadias [5]. 

A prostatic utricle cyst can have varied presentation ranging from asymptomatic to recurrent UTI, epididymitis, hematuria, pyuria, urinary incontinence, oligospermia, retention or constipation [1, 4]. Rarely it may enlarge and present as an abnormal lower abdominal or pelvic mass or observed during pelvic or trans-rectal ultrasonography as a fluid filled cavity between bladder and rectum [5, 6]. Though MRI can easily identify these cysts by virtue of their high signal on T2-weighted images [7], simpler radiological studies like retrograde urethro¬graphy can equally differentiate utricle cysts from other prostatic and periprosatic cysts demonstrating it location and connection with posterior urethra [8]. Endoscopic cannulation of the utricular opening with contrast material infusion into the cyst is another method to accurately demonstrate the communication of the urethra with the mass, and can determine the anatomical relationship with surrounding structures [1]. Because these conditions are usually managed conservatively, pathologic proof is not possible in all cases, and the diagnosis is often made on the basis of clinical features and imaging appearance [7].

Differential diagnoses include mullerian duct cysts, bladder diverticulum, teratoma, seminal vesicle cyst, epididymal cyst and Wolffian duct cyst [1, 6]. Mullerian duct cyst though may be connected to the verumontanum by a stalk; it does not communicate with the posterior urethra, present late in life and is not associated with genitourinary anomalies [1, 7]. Seminal vesicle and Wolffian duct cysts are located off the midline and contain sperms [1]. Seminal vesicle cysts result from congenital atresia of the ejaculatory duct and are often associated with ipsilateral renal agenesis [7].

Surgical management of prostatic utricle cyst remains challenging, because of the rarity of this disorder and due to the close proximity of these lesions to the ejaculatory ducts, pelvic nerves, rectum, vas deferens and ureters [1]. Schuhrke et al, reported endoscopic transurethral cyst catheterization and aspiration, cyst orifice dilatation, incision, or unroofing that suits for small prostatic utricle cysts but recurrence rates were high [3]. Open excision is the better definitive treatment but the problem is the location of cyst which is considered too high to be approached through the perineum or too low to be approached through the abdomen. Several approaches have been described. However, all require extensive dissection, sometimes having two stages and often result in poor exposure. The efforts sometimes ended in incomplete excision, and complicated dissection often needed the excision of one or both seminal vesicles, vas, and portions of the prostate [1, 4].

Surgical treatment should be reserved for symptomatic utricle cysts only. Clinical dilemma arises when a patient presents with symptoms. Concomitant presence of other pathologies may produce similar symptoms and treatment of primary pathology should be done first. Chances of associated infer¬tility and incontinence must be explained to the patient before intervention [8]. In our case, the prostatic utricle was asymptomatic and the presenting symptoms were due to the bulbar urethral stricture. Thus surgical treatment should be individualised and offered to those with persistent symptoms where other causes of similar symptoms have been are treated.

## Footnotes

**Source of Support:** Nil

**Conflict of Interest:** None declared

